# A scoping review of the impact of long-distance truck drivers on the spread of COVID-19 infection

**DOI:** 10.11604/pamj.2021.38.27.26691

**Published:** 2021-01-12

**Authors:** Thobile Malinga, Charles Shey Wiysonge, Duduzile Ndwandwe, Joseph Chukwudi Okeibunor, Ambrose Otau Talisuna

**Affiliations:** 1 Cochrane South Africa, South African Medical Research Council, Cape Town, South Africa,; 2 Department of Global Health, Stellenbosch University, Cape Town, South Africa,; 3 School of Public Health and Family Medicine, University of Cape Town, Cape Town, South Africa,; 4 World Health Organization Regional Office for Africa, Brazzaville, Congo

**Keywords:** Long-distance truck drivers, pandemic, prevention, coronavirus disease

## Abstract

**Introduction:**

long-distance truck drivers have been identified as a high-risk group for coronavirus disease (COVID-19) infection. Thus, the aim of this scoping review is to map out the existing evidence on the impact of long-distance truck drivers on the spread of COVID-19 and measures that countries can implement to mitigate this route of spread in the African region.

**Methods:**

we searched the PubMed database and the website of the World Health Organization (WHO) in March 2020 for eligible studies.

**Results:**

the search strategy identified 669 citations, of which six met the inclusion criteria. The most frequently reported interventions were maintaining hand hygiene, social distance, testing truck drivers, regulation of trade and transport e.g. only trucks with the food, medical supplies, fuels, agricultural supplies will be allowed to operate in interstate operations and regulating and controlling trucks carrying essential goods and services e.g. truck drivers are required to declare their final destination and are urged to stop only at designated points. Two studies from the African region reported about border closures and entry and exit screening, two studies from the US reported about the threat for public safety and risks and mitigation plans and 2 guidelines reported about harmonisation and facilitation of cross border in the context of the COVID-19 outbreak.

**Conclusion:**

this review highlights the countries response to mitigate the impact of the pandemic by implementing measures to facilitate safe cross-border trade and adopting regional harmonization of trucking regulations.

## Introduction

COVID-19 confirmed cases have been reported globally [1]. The first case in Africa was confirmed in Egypt on February 14^th^, 2020 [2,3]. COVID-19 has led to the introduction measures meant to prevent the spread of the virus, including testing and waiting for the test results for truck drivers. According to [4] public health events can cause serious crises and damage to the human population if effective surveillance systems; and programmes to prevent and respond promptly to health threats. The recent West-African ebola outbreaks and the zika virus have highlighted considerable room for improvement in meeting the imperative to research and rapidly develop effective strategies to curb the spread of the virus [5]. It was difficult to bring under control owing to high infectivity, weak health systems, rampant fear and mistrust among the affected population and fluid cross-border movement of peoples [6]. Thus, screening measures of truck drivers at the point of entry including ports can be implemented to prevent intercontinental transmission of disease by early detection.

Long-distance truck drivers around the world have been identified as a high-risk group for this pandemic and are consequently the targets for prevention and education-based intervention. While most of these interventions have addressed individual-level risk behaviour, more attention needs to be paid to the structural barriers e.g. social disruption due to COVID-19 pandemic of truck drivers [7]. Fears that are associated with the current global spread of the novel coronavirus disease have trickled into almost every industry including the trucking industry as well. Some companies try their best to ensure that the drivers are healthy, rested and fed during the COVID-19 outbreak, but this is not always the case. Concerns like a lack of medical facilities for drivers who may develop symptoms while on the road may fuel the spread of the virus because truck drivers are part of mobile populations which have been noted as a key population at risk [8].

In some countries of the African region, transporting of goods is essential for the economy and the trucking industry play a major role. Due to the transcontinental nature of the transport industry, health plan of action to prioritize truck drivers need complementary national healthcare policies. Research has demonstrated that most countries in the African region are aware of the vulnerability of truck drivers to poor health outcomes and the member states have developed strategic plans to address such issues [9]. Each country´s functional capacity to manage health security issues is based on WHO International Health Regulations (IHR) 2005 which states the WHO recommendations in response to a Public Health Emergency of International Concern (PHEIC) may include screening measures at points of entry, Monitoring and Evaluation Framework (MEF) and on an indicator of vulnerability to emerging epidemics [4]. Thus, following this the South African Development Community (SADC) developed guidelines on harmonisation and facilitation of cross border transport operations across the region during the COVID-19 pandemic as a measure to curb the spread of the virus within the region [10]. However, further planning for efficient implementation, scale-up and the ability to maintain healthcare programmes for truck drivers is stalled by knowledge gaps within this population and the impact of existing healthcare services on health outcomes [9]. Hence the need to conduct this scoping review.

## Methods

**Figure 1:**
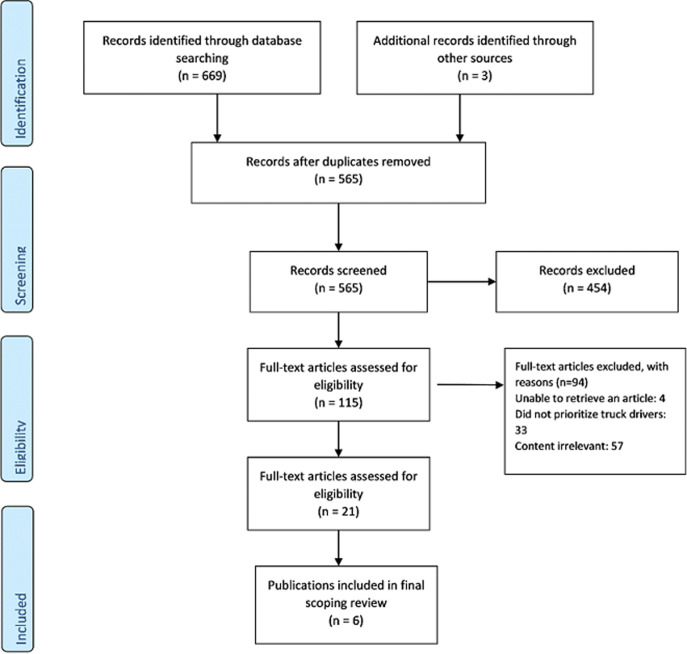
flow diagram showing the seach and selection of studies for the review

The authors performed a scoping review of the published and unpublished literature. We adhered to the PRISMA ScR guidelines for reporting [11] of scoping review, see the PRISMA flow diagram (Figure 1).

**Eligibility criteria:** the studies were selected according to population-concept-context (PCC) framework recommended by Joanna Briggs Institute [12] for scoping reviews. Participants: truck drivers; concepts: health promotion; health interventions; context: countries and borders within the African region.

**Search strategy:** the authors searched scientific databases such as PubMed and WHO website and country level-reports (SADC), using a broad search strategy including all fields and free text. The search included the following word combinations: “long distance” or “long distance” or “cross-border” or “cross-border” or “international” or “regional” and “truck” or “transport” or “transportation” or “driver” or “drivers” and “COVID-19” or “COVID” or “corona” or “coronavirus” or “SARS-CoV-2” or “SARS-CoV-2” and “pandemic influenza” or “H1N1” or “MERS” or “SARS”. Research was conducted on studies and reports published between 2014-2020. A detailed search strategy is provided in Table 1. The review strategy had been shaped using the peer review of electronic search strategies (PRESS) 2015 guideline statement, which was helpful to guide and improve the peer review of electronic literature search strategies [13].

**Table 1:** search strategy

	Search string
#1	Long distance or long-distance or cross-border or cross border or international or regional
#2	Truck or transport or transportation or driver or drivers
#3	#1 and #2
#4	COVID-19 or COVID or corona or coronavirus or SARS-CoV-2 or SARS-CoV 2
#5	Pandemic influenza or H1N1 or MERS or SARS
#6	#4 and #5
#7	#3 and #6

**Study selection:** the lead author TM first examined the titles and abstracts were and after discussion with CW an agreement was reached to include all publications and reports describing a healthcare programme in the African region providing services designed specifically for truck drivers. The full text records of all the material selected for further examination were assessed for inclusion. The author TM confirmed the final list of selected material with CW.

**Extraction and charting the results:** data extraction forms were created for both primary studies and reports and the coded into structured categories according to: author(s); year of publication; country of focus; and key message that relate to the review question (Table 2). Figure 1 displays the flow chart as to the database search and final article selection. Data were then summarized using narrative description to outline the characteristics of the included studies.

**Table 2:** summary of evidence for included studies for addressing the impact of long-distance truck drivers on the spread of COVID-19 infection

Author (year)	Region of focus	Type of document	Key message
Nakkazi (2020)	East Africa	Commentary	The author made no specific conclusion regarding interventions (for impact of drivers for prevention of COVID-19) but highlighted that with disparities in the response to COVID-19 in many African countries the authorities are trying to isolate sources of infections; for example, Tanzania's neighbors, they perceived truck drivers to be a source of new cases, as a result, Kenya, Uganda and Zambia have introduced border closures and tighter preventive measures on truck drivers' movements
Baveja (2020)	US	Journal article	As with any plan, there is a need to be cognizant of its risks and challenges; every administrative zone will encounter risks and develop mitigations plans that are most appropriate for its situation; the authors strongly recommend setting up best-practice sharing platforms that will help disseminate effective ideas amongst communities
Lemke (2020)	US	Commentary	Targeted research and prevention-oriented action steps are urgently needed to curb the likelihood of the development of this pandemic, a network of health problems, ones that share common social underpinnings and cause an increased public health burden on a community (poses an imminent, significant and widespread threat for public health and safety), especially as mandated physical distancing policies heterogeneously expire, the American economy gradually reopens and freight volumes increase-along with US long-haul truck drivers' vulnerability to acquiring and transmitting COVID-19
Mouchtouri (2019)	West Africa, Canada, Singapore, Australia	Journal article	Highlights lessons learned from previous pandemics like Ebola, H1N1, SARS that little evidence is available about entry and exit screening measure implementation and effectiveness at ports and ground crossings; for preparedness purposes and to be ready to respond to any unexpected public health event, all countries should have the capacities to implement entry and exit screening at designated ports, airports and ground crossings; exit screening measures could be prioritized compared to entry measures, based on past temporary recommendations issued during Public Health Emergency of International Concern (PHEIC)
Fernandez (2020)	SADC region	Guideline	The member states and other key stakeholders and partners should: review national transport related policies, regulations and response measures and identify inconsistencies; based on the review, select best practices and propose to the member states harmonized policies, regulations and measures; put in place a mechanism to enable member states to share information on COVID-19 response policies, regulations and guidelines, best practices and experiences in the implementations of the various measures
WHO (2020)	Global including Africa	Guideline	Points of entry should adhere to the following guidance: wear a tightly fitted medical mask that covers the nose and mouth when entering all the time when they are dealing with people including truck drivers clean their hands with alcohol-based hand rub or soap and water after handling any equipment or documents

## Results

**Selection of sources of evidence:** search results yielded 669 articles in PubMed 3 from WHO website. After removal of duplicates, 565 records remained. Out of these, 115 titles were screened for relevant studies, 21 abstracts were then scanned for acceptable inclusion criteria. Finally, 6 full text articles were included in the review. Several studies were excluded due to lack of focus on the study topic, lack of reported issue on truck drivers and no new evidence was demonstrated by the studies.

**Characteristics of sources of evidence:** a total of 6 articles (2 guidelines, 2 commentaries and 2 journal articles) were included in our final analysis. The most frequently reported interventions were maintaining hand hygiene, maintaining social distance and testing truck drivers, regulation of trade and transport and regulating and controlling trucks carrying essential goods and services. Two studies from the African region reported about border closures and entry and exit screening, two studies from the US reported about the threat for public safety and risks and mitigation plans.

**Synthesized results:** the peer-reviewed literature on the impact of long-distance truck drivers on the spread of the virus and measures that countries can implement to mitigate this route of spread of COVID-19 is recent and it limited. Among the included studies, 83% were published in the past 5 months of the current review (2020). Most of the studies were mainly from the African region. Out of the 6 included studies, 3 studies were focused on low-income countries, 2 studies in high-income countries and 1 in both low- and middle-income countries. The included studies in the review have used various types of study design: n=2 guidelines, n=2 commentaries and n=2 journal articles (Table 1). However, it was difficult to identify the study approach because the authors did not provide enough details on their methodology.

One guideline addressed harmonisation and facilitation of cross border transport operations across the region during the COVID-19 pandemic and the other document addressed the management of ill travellers at points of entry (international airports, seaports and ground crossings) in the context of COVID-19. The member states within the Southern African Development Community (SADC) region have come up with guidelines to deal with the impact of COVID-19 in the African region. These includes: regulation of trade and transport: stating that those trucks that carry goods will be allowed to operate in interstate operations to ensure continuity of supply chains including and not limited to food, medical supplies, fuel, humanitarian relief services and other goods and products as may be agreed among member states.

Regulating and controlling trucks carrying essential goods and services: the member states should ensure that national policies, regulations and guidelines to ensure that: law enforcement officers treat with leniency and recognize the extension of validity of short-term cross border permits/licenses, which have expired en-route due to different administrative procedures in force following the COVID-19 measures until the vehicles can complete the journey. Second, guidelines should recommend that vehicles only have 2-3 crew members per vehicle to facilitate smooth border crossing in the region. Thirdly, if a driver or crew member is showing signs for COVID-19, the truck will be de-contaminated before it is allowed to continue to its final destination and the driver or crew member must be referred to a treatment center at operator´s cost. Finally, truck owners should make necessary arrangements for a backup if the crew is quarantined while in transit and truck drivers should declare their final destination and urged to stop only at designated points along the transport corridors [3,10].

Lockdown regulations on truck drivers and communities: among the studies that reported findings and outcomes related to lockdown regulations on truck drivers and communities. They made no specific conclusion regarding interventions (for the impact of drivers for prevention of COVID-19) but highlighted that with disparities in the response to COVID-19 in many African countries the authorities are trying to isolate sources of infections. For example, Tanzania´s neighbours, they perceived truck drivers to be a source of new cases. As a result Kenya, Uganda and Zambia have introduced border closures and tighter preventive measures on truck drivers´ movements [14]. Therefore, to counteract the outcome tracing internal community infections and contacts is of paramount importance and reducing the time of getting the results for truck drivers.

Long-distance truck drivers and physical distancing, provision of personal protective equipment, screening programme and communities: two studies explored key COVID-19 metrics that need to be established for this population. Relationships between long-haul trucker network attributes and COVID-19 spread need to be delineated. Furthermore, interactions between endemic health disparities and COVID-19 vulnerability need to be elucidated and policies and interventions need to be identified and implemented [15,16] reported that stopping all international, domestic passenger air and intercity bus/train travel; create administrative zones of about 1 million people; stop all non-emergency cross-zonal travel except for transportation of goods, deploy an information-driven service value chain to control the spread of the pandemic within a zone might assist to curb the spread.

Entry and exit screening, contact tracing, laboratory testing of suspected cases: the authors in this study highlighted lessons learned from previous pandemics like Ebola, H1N1, SARS that little evidence is available about entry and exit screening measure implementation and effectiveness at ports and ground crossings. For preparedness purposes and to be ready to respond to any unexpected public health event, all countries should have the capacities to implement entry and exit screening at designated ports, airports and ground crossings. Exit screening measures could be prioritized compared to entry measures, based on past temporary recommendations issued during PHEIC [4].

## Discussion

This scoping review used standard systematic review methods to identify, select and synthesize findings from 6 studies that reported impact of long-distance truck drivers on the spread of the virus and measures that countries can implement to mitigate this route of spread. Early detection of COVID-19 and prevention of onward transmission are crucial challenges to all countries in the African region [2]. Onward spread possibly occurring in countries with weaker health systems is a major public health concern [14]. Some countries remain ill-equipped. Some are without the diagnostic capacity for rapid testing for the virus, which might critically increase the delay from the identification of suspected cases to their confirmation and isolation, affecting possible disease transmission [17]. World Health Organization (WHO) is currently supporting countries to improve their diagnostic capacity.

This scoping review has demonstrated that in the African region viruses like COVID-19 may be transmitted by people who work along the roads like truck drivers. Truck drivers have become a core group for COVID-19 and they have generated significant local transmission, which now threatens a full-blown epidemic unless strict controls are put in place [18]. For example, since these drivers cannot have a single home, they establish a semi-permanent home at every stop exposing everyone who they come into contact with. During the COVID-19 lockdown, the drivers are the ones who´ve been carrying the food, the medical supplies and every other essential item designated under the regulations. Unlike petrol attendants, farmworkers, supermarket cashiers and others who get to go home at the end the day, truck drivers spend days, if not weeks, away from their families as they continue to serve the nation during this crisis time. However, this could lead to a major virus epidemic and researchers have started to study ways by which the virus might continue to enter countries and how it might spread. An observation of long-distance truck drivers demonstrates that there might be a relationship between highways and the spread of COVID-19.

**Limitations:** our review presents several limitations. Firstly, we aimed to map out the evidence on the measures that countries can implement to mitigate prioritizing truck drivers based on available information. Owing to the nature of the documents reviewed, we were unable to assess the quality of methodology that was used. Moreover, we focused on reports, studies and guidelines available publicly. Due to the nature of the documents reviewed, we were unable to assess the quality of services provided in these programmes. Lastly, is that new data are added daily, the pandemic is dynamic. Data analysis should be done on a continuous basis to obtain a completer and more up-to-date picture.

## Conclusion

As COVID-19 continue to spread globally including the African region research should be considered to assess the impact on all possible prevention measures. This review illustrates a global picture of the current academic literature on the impact of long-distance truck drivers and the spread of COVID-19. The inconsistency and the lack of strong quality in the methodology of the included studies are proof that more rigorous studies are needed to demonstrate the positive and adverse impact of long-distance truck drivers during the COVID-19 pandemic. Nevertheless, the existing evidence from this review highlights that following the WHO guidelines can assist to curb the spread of COVID-19. Furthermore, these studies should look at how do the infected individual´s age and other characteristics of a case (i.e. patient) affect the risk of transmitting the infection to others. Primary studies to evaluate the effect of truck drivers and the spread of COVD-19 are still needed. The most promising intervention seems to be to educate the drivers ineffective strategies like frequent handwashing. Also, collaborative efforts among trucking services and governments are important to ensure that the proposed guidelines are adhered to. Without these measures, truck drivers will be left behind. Screening should be part of the government´s recommendations in certain points of entry for specific periods. Furthermore, screening for COVID-19 in the most affected African countries could assist to identify cases. Future research should also include economic evaluations as well as methods to determine facilitators and barriers to programme participation and continuation.

### What is known about this topic

- Very few data are available regarding the impact of long-distance truck drivers on the spread of the virus and measures that countries can implement to mitigate this route of spread;
- Truck drivers drive long distances, exposing themselves to a more extensive social network, primarily urban and likely crowded places such as ports where the probability of contact with infected person may be increased;
- Some countries in the region remain ill-equipped. Some are without the diagnostic capacity for rapid testing for the virus, which might critically increase the delay from the identification of suspected cases to their confirmation and isolation, affecting possible disease transmission.

### What this study adds

- For preparedness purposes and to be ready to respond to any unexpected public health event, all countries should have the capacities to implement entry and exit screening at designated ports, airports and ground crossings;
- Public health prevention measures that take into account regional integration of efforts are required to ensure success for the COVID-19 programs for truck drivers.
